# Full-endoscopic removal of third ventricular colloid cysts: technique, results, and limitations

**DOI:** 10.3389/fsurg.2023.1174144

**Published:** 2023-06-02

**Authors:** Tugrul Cem Unal, Altay Sencer, Ilyas Dolas, Cafer Ikbal Gulsever, Duran Sahin, Duygu Dolen, Musa Samet Ozata, Metehan Ozturk, Yavuz Aras, Aydin Aydoseli

**Affiliations:** Department of Neurosurgery, Istanbul Faculty of Medicine, Istanbul University, Istanbul, Turkey

**Keywords:** colloid cyst, full-endoscopic surgery, hydrocephalus, neuroendoscopy, swiveling technique

## Abstract

**Introduction:**

Colloid cysts (CCs) are rare benign lesions that usually arise from the roof of the third ventricle. They may present with obstructive hydrocephalus and cause sudden death. Treatment options include ventriculoperitoneal shunting, cyst aspiration, and cyst resection microscopically or endoscopically. This study aims to report and discuss the full-endoscopic technique for removing colloid cysts.

**Materials and methods:**

A 25°-angled neuroendoscope with an internal working channel diameter of 3.1 mm and a length of 122 mm is used. The authors described the technique of resecting a colloid cyst by a full-endoscopic procedure and evaluated the surgical, clinical, and radiological results.

**Results:**

Twenty-one consecutive patients underwent an operation with a transfrontal full-endoscopic approach. The swiveling technique (grasping the cyst wall and rotational movements) was used for CC resection. Of these patients, 11 were female, and ten were male (mean age, 41 years). The most frequent initial symptom was a headache. The mean cyst diameter was 13.9 mm. Thirteen patients had hydrocephalus at admission, and one needed shunting after cyst resection. Seventeen patients (81%) underwent total resection; 3 (14%), subtotal resection; and 1 (5%), partial resection. There was no mortality; one patient had permanent hemiplegia, and one had meningitis. The mean follow-up period was 14 months.

**Conclusion:**

Even though microscopic resection of cysts has been widely used as a gold standard, successful endoscopic removal has been described recently with lower complication rates. Applying angled endoscopy with different techniques is essential for total resection. Our study is the first case series to show the outcomes of the swiveling technique with low recurrence and complication rates.

## Introduction

Colloid cysts (CCs) are rare benign lesions constituting approximately 0.2%–2% of all brain tumors. They usually arise from the roof of the third ventricle near the foramen of Monroe, although other locations have also been reported (1). Histopathological research indicates that endodermal tissues have been misperceived as the origin of CC. Colloid cysts usually have a thin collagen wall containing largely mucinous material with different densities. The proximity to the foramen of Monroe of these otherwise benign lesions can lead to obstructive hydrocephalus and sudden death in some rare cases (2). Therefore, surgical resection is the treatment of choice for large (>1 cm) symptomatic cysts obstructing the foramen of Monroe and causing hydrocephalus (3).

Options for surgical treatment include ventriculoperitoneal shunting, aspiration of cyst material, microsurgical resection of the CC, and, more recently, endoscopic resection. Currently, cerebrospinal fluid (CSF) shunting without resection is frequently abandoned. Simple aspiration of cyst material without removal of the cyst wall is also avoided owing to the likelihood of recurrence. Although microsurgical removal through transcortical (with or without the use of tubular retractors) approaches has been used widely, with the increasing use of endoscopic techniques, successful endoscopic removal of CCs has also been reported. Different techniques for accessing and removing CCs are discussed in the literature (4, 5). Some surgeons prefer a tubular retractor with endoscopic assistance instead of a full-microscopic or full-endoscopic approach (6, 7). Others use the full-endoscopic technique and perform the entire operation through the endoscope's working channel (8). Dorsch et al. described the swiveling technique for the endoscopic removal of CCs; however, their study was a technical note performed on one patient (9). There was a need for a study with more cases to delineate whether swiveling technique reduces the complication rates and the rate of remnant cyst capsule compared to microscopic and conventional endoscopic approaches.

In this study, we aim to describe the full-endoscopic technique for removing third ventricle colloid cysts and report the results of a single institution experience.

## Materials and methods

### Patient population

This study was performed by the ethical standards of the Institutional Review Board of Istanbul University, Faculty of Medicine. The data of 21 patients who underwent an operation at Istanbul University, Faculty of Medicine, Department of Neurosurgery between 2008 and 2019 by the same surgical team using the full-endoscopic approach were evaluated retrospectively. The patients were offered surgical treatment because they had either hydrocephalus (with or without clinical symptoms) or headache that could be attributed to intermittent obstruction of the foramen of Monroe. The series was consecutive, and there was no microsurgically treated patient for the initial surgical option. The patient's preoperative and postoperative clinical statuses and magnetic resonance imaging (MRI) scans were evaluated.

### Surgical instruments

The 25°-angled endoscope has an outer diameter of 5.9 mm. The endoscope contains a 3.1-diameter eccentric working channel, the light conductor system, a channel for continuous irrigation, and the optical lens system. The working sheath has an outer diameter of 6.9 mm. Scissors, forceps, and dissector are introduced from the working channel. All the surgical instruments and optics were used from WOLF (Richard Wolf GmbH, Knittlingen, Germany).

### Operative technique

**Figure 1:**
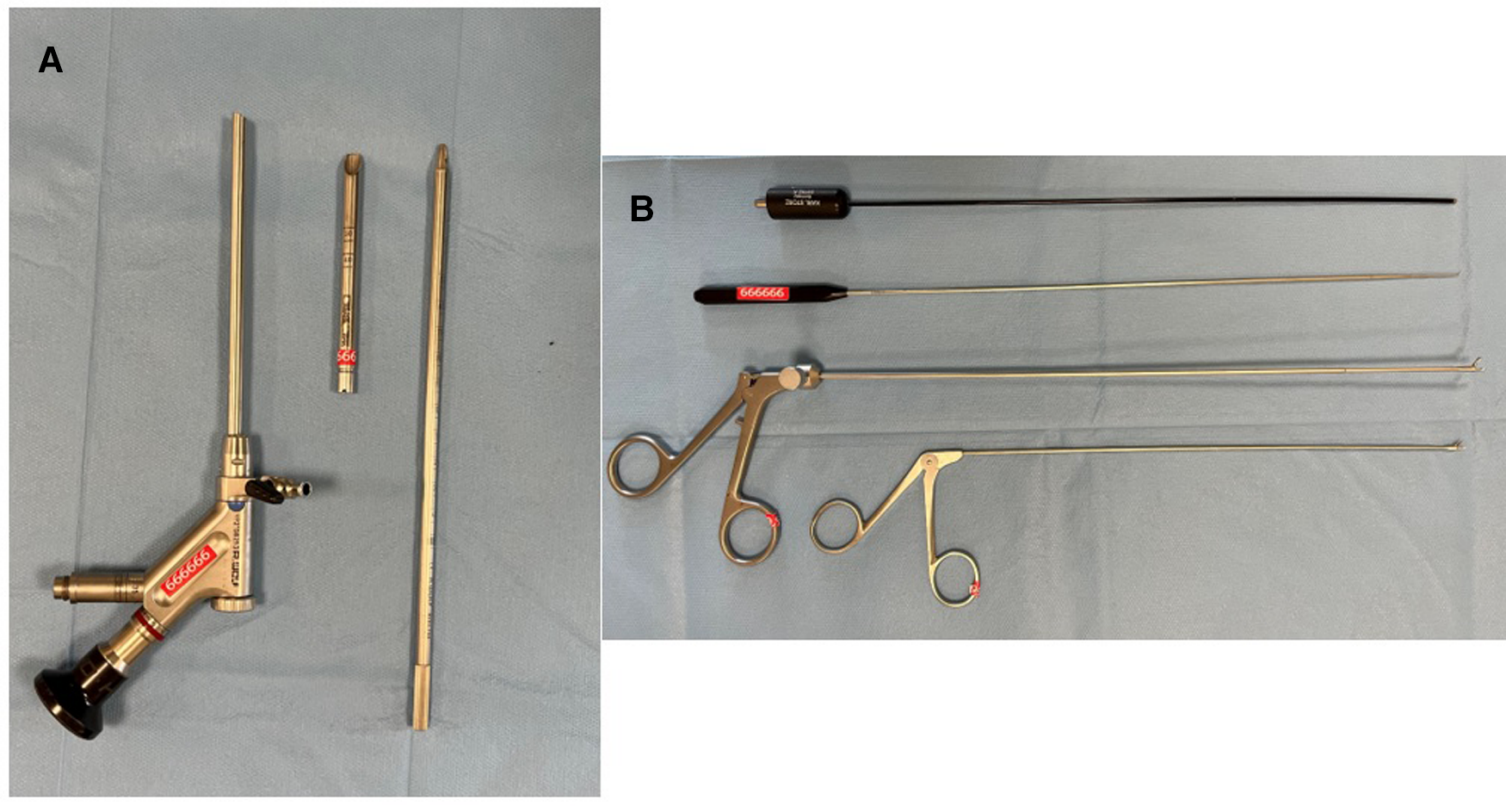
The working sheath, the trocar, and the 25°-angled endoscope with a working channel diameter of 3.1** **mm and a length of 122** **mm were used (**A**). During the procedure, bipolar cautery, scissors and forceps are introduced through the working channel (**B**).

A large burr-hole approximately 14 mm in diameter was made 5 cm anterior to the coronal suture and 4 cm lateral to the midline. We believe that entering the lateral ventricle from this location allows an adequate view of the third ventricle cyst. Usually, the side with the larger ventricle was chosen, and if both ventricles were equal, the right side was used to remain in the non-dominant hemisphere. Ventricular puncture is assisted by intraoperative ultrasonography or a neuronavigation system for accurate access even to slit ventricles. A trocar introducer is used for ventricular puncture. After entering 5–6 cm and visualizing CSF flow, the trocar was removed, and the sheath was stabilized with the aid of the assistant. The neuroendoscope system with an internal working channel diameter of 3.1 mm and a length of 122 mm is inserted through the sheath (Figure 1). Once the 25°-angled neuroendoscope is introduced to the lateral ventricle, the surgical procedure is performed under visual control.

**Figure 2:**
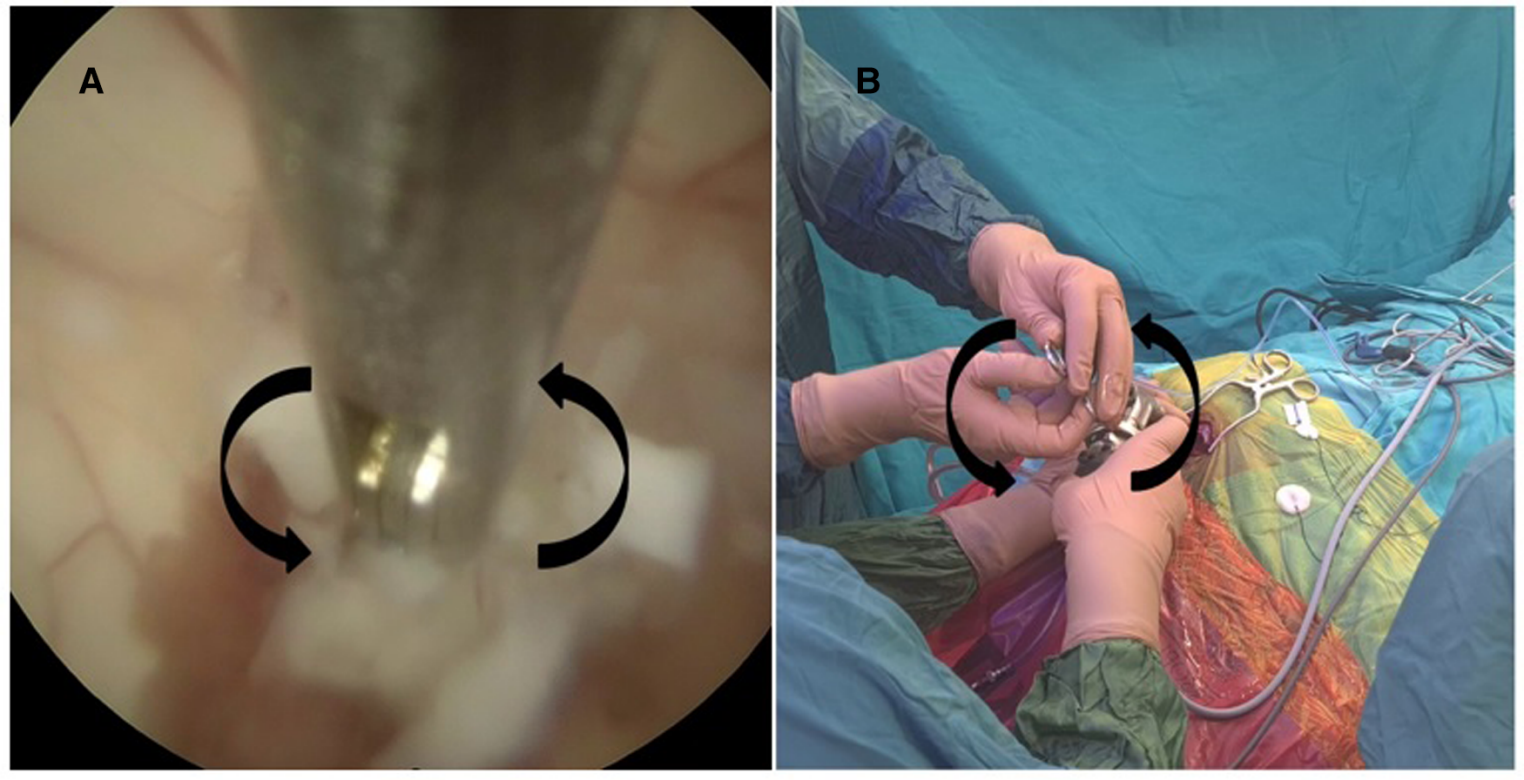
The swiveling technique is seen in the endoscopic view (**A**) and from the surgeon's hands (**B**).

The cyst can usually be identified at the level of the foramen of Monroe. We prefer to make a small incision on the cyst and empty the content using a pediatric-size (8-F) suction cannula (Supplementary Video S1). We prefer to aspirate the cyst components before resecting the cyst wall. Resection of the cyst without aspirating its components may result in cyst rupture, which may impair vision and cause a subtotal resection of the remaining cyst wall. In some cases, the cyst can be located posteriorly in the third ventricle. In these cases, a small incision parallel and medial to the choroid plexus may also be necessary. After emptying the cyst contents and mobilizing the cyst, the attachments of the cyst are coagulated. Firm grasping of the cyst wall with small forceps and rotational movements (swiveling technique) (Figure 2) provides total resection of the cyst wall (Supplementary Video S1) (9). Bleeding can be controlled by irrigation, coagulation, or balloon pressure. If inadequate hemostasis is suspected, a ventricular catheter may be placed.

### Postoperative period

On the postoperative first day, the first and sixth-month MRIs were obtained in the follow-up period. A specialized neuroradiologist evaluated the extent of colloid cyst resection and hydrocephalus status. Total resection was defined when even the cyst capsule was not detectable in the images. A routine external ventricular drainage (EVD) was not left after the surgery for evaluating CSF diversion dependency. The patients were transferred to the neurosurgical floor service after the surgery. There was no need for monitoring in the intensive care unit except for the patient with uncontrolled bleeding with conversion to the microsurgical technique.

## Results

**Figure 3:**
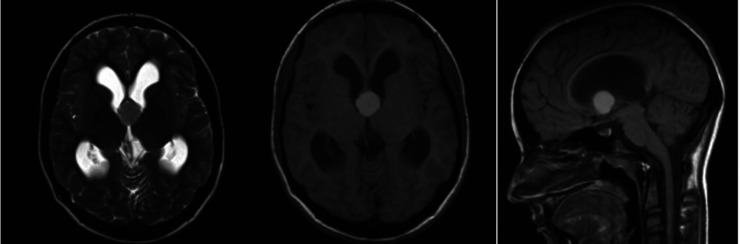
Colloid cyst causing hydrocephalus.

Twenty-one patients underwent the transfrontal full-endoscopic approach. Of the patients, 11 were female, and ten were male, aged 19–65 years (mean age, 41 years). The most frequent initial symptom was headache (57%), followed by nausea and vomiting (29%). Eight patients had signs of increased intracranial pressure (Table 1). The cyst diameter was 6 to 22 mm (mean, 13.9 mm) on the MRI scans. Hydrocephalus was observed in 13 patients (62%); the MRI scan of a patient with hydrocephalus can be seen in Figure 3.

**Table 1:** Patients’ characteristics.

# Patients	Gender	Age	Complaint	Duration of complaint (days)	T1W	T2W	Cyst diameter (mm)	Extent of resection	Hydrocephalus (+/−)	Follow-up period (months)
1	F	19	H/A	30	Iso	Hyper	11	Total	+	2
2	F	35	N/V, H/A	10	Hyper	Hyper	12	Total	−	24
3	M	65	N/V	90	Hyper	Hyper	18	Total	+	14
4	F	38	UI	1,095	Iso	Hyper	22	Total	+	14
5	M	21	H/A	180	Hypo	Hyper	6	Total	−	15
6	M	57	H/A	22	Hyper	Hyper	15	Total	+	13
7	F	46	H/A	21	Hyper	Hyper	17	Total	+	5
8	F	52	H/A, Blurred vision	14	Iso	Iso	14	Total	−	18
9	F	64	H/A	120	Hyper	Hyper	19	Subtotal	+	16
10	M	26	Altered mental status	1	Iso	Hyper	12	Total	−	14
11	M	59	N/V, H/A	5	Hyper	Hyper	16	Total	+	15
12	F	49	H/A	49	Iso	Hyper	20	Total	+	14
13	M	32	N/V	4	Hypo	Hyper	15	Subtotal	+	22
14	F	28	Blurred vision	7	Hyper	Hyper	16	Total	+	18
15	F	39	Altered mental status	2	Iso	Iso	7	Total	−	4
16	M	24	H/A	240	Hyper	Hyper	18	Partial	−	21
17	M	47	UI	90	Iso	Hyper	11	Total	+	24
18	M	37	H/A	730	Hyper	Hyper	10	Total	−	9
19	F	38	N/V	21	Iso	Hyper	5	Total	−	13
20	F	39	H/A	30	Hyper	Hyper	18	Subtotal	+	13
21	M	46	N/V	14	Iso	Iso	10	Total	+	5

F, female; M, male; H/A, headache; N/V, nausea and vomiting; UI, urinary incontinence; T1W, T1-weighted magnetic resonance imaging (MRI); T2W, T2-weighted MRI.

**Figure 4:**
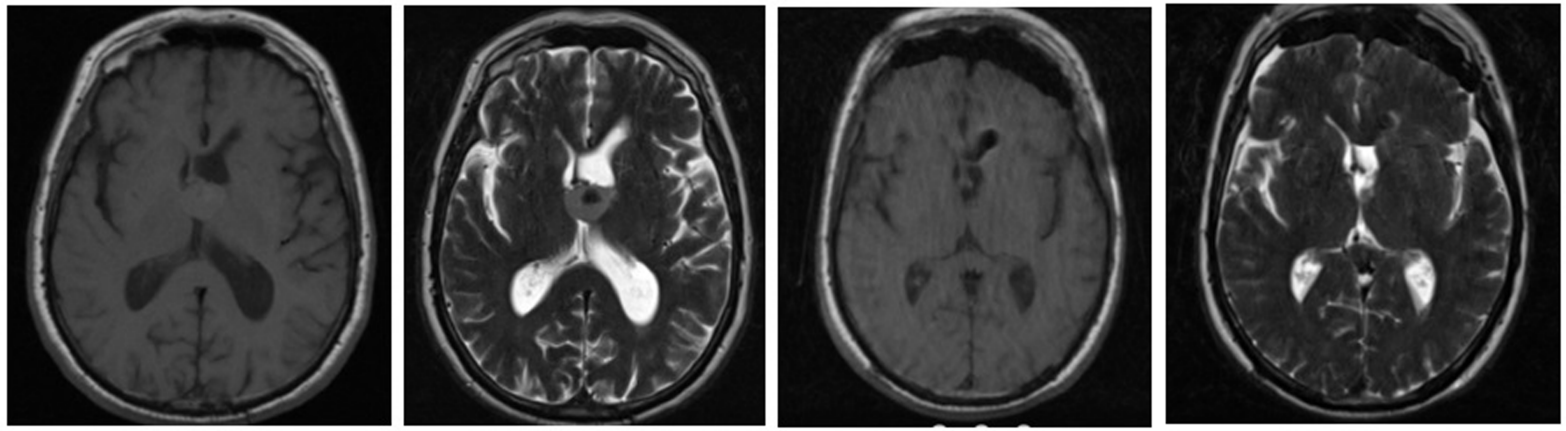
Preoperative and postoperative T1- (T1W) and T2-weighted (T2W) axial images of a patient who underwent total resection.

**Figure 5:**
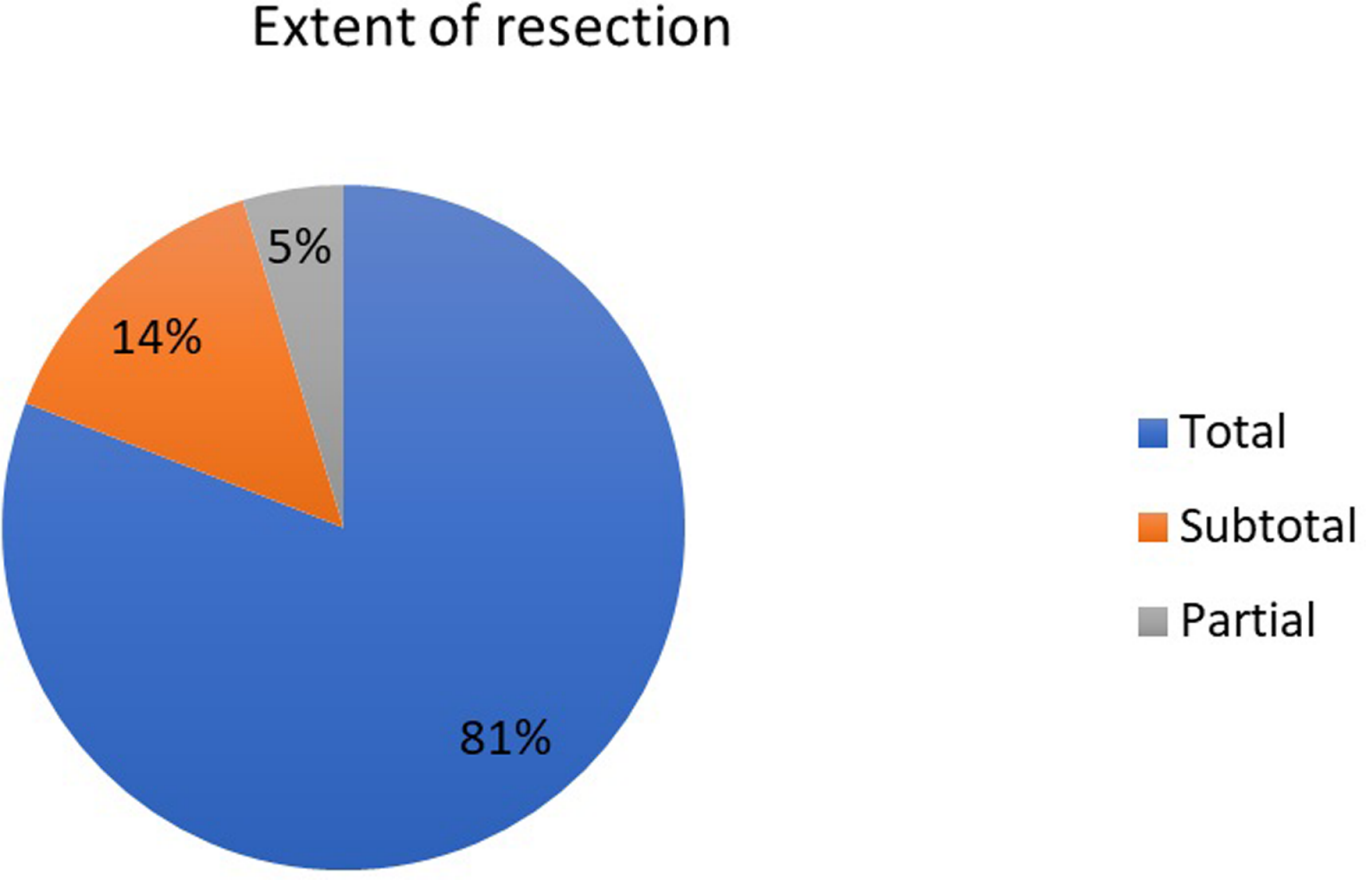
Extent of resection.

Total resection was achieved in 17 patients (81%). A preoperative and postoperative MRI scan is shown in Figure 4. Postoperative image shows total removal of the cyst. Subtotal resection was seen in 3 patients (14%). The extent of resection was defined by a specialized neuroradiologist by examining the early postoperative MRI scans (24–48 h). The distribution of the patients according to the extent of resection can be seen in Figure 5. The histopathological diagnosis of CC was confirmed in each patient.

In all the patients, the preoperative symptoms resolved rapidly. Minor morbidity (a case of postoperative meningitis) and major morbidity (a case of permanent hemiplegia) were observed. For the patient with postoperative hemiplegia with uncontrolled bleeding, the surgery was converted to microsurgery. The same patient required a ventriculoperitoneal shunt in the follow-up period. One patient had a short-term memory deficit, probably due to the fornix injury, which improved in 6 months. There was no mortality. The postoperative follow-up periods ranged from 2 to 24 months (mean follow-up period, 14 months). During the follow-up period, first and sixth-month MRI scans were performed. No recurrences were observed during the follow-up period (total or subtotal resection), hence no reoperation. Of 13 patients that presented with hydrocephalus, only one required the CSF diversion method, and the others had resolved hydrocephalus after the removal of the CC.

## Discussion

Considering the published 10% risk of sudden death, symptomatic (according to some authors, asymptomatic) CCs warrant surgical intervention (10, 11). The best approaches for the surgical treatment of CCs are controversial. Techniques such as ventriculoperitoneal shunting or cyst aspiration by stereotactic procedures are frequently abandoned in modern neurosurgery. Stereotactic cyst aspiration is a minimally invasive, safe, and effective procedure, but it only provides the removal of cyst material. Dense or small cysts can cause problems. The cyst capsule is left behind, causing high recurrence rates (12). As stated in a meta-analysis, microsurgical excision and endoscopic removal are the most commonly applied surgical methods. The choice between the methods is primarily based on the surgeon's expertise and preference (13–16).

### Technique

The aim of microsurgical procedures is total resection of the CC, including the capsule. Microsurgical resection using the transcallosal or transcortical frontal approach (with or without tubular retractors) allows excellent visualization and bimanual manipulation of the cyst (17). However, with the advanced use of endoscopy in cranial and spinal procedures, endoscopic resection of CCs has gained widespread use as a less invasive technique after it was first reported in 1983 (18). To our knowledge, even though there are multiple studies on the endoscopic technique for colloid cyst removal (1, 3, 17), there is only one study that describes the swiveling technique (9). The common approach in resecting the colloid cyst is to aspirate its content and coagulate the cyst wall to shrink it. In order to open the cyst wall, we used bipolar cautery, then we incised the cyst wall with microscissors, which were adequate for all the patients. However, in some studies, lasers such as fiberoptic neodymium-yttrium garnet (Nd-YAG) are shown to be effective in opening the cyst wall. The laser was also used for septostomy if preferred. Then dissecting the cyst wall with the cautery and coagulation of its small feeders are performed (1, 3, 17, 19). In our technique, we apply a similar approach, but we tend to leave the weak adhesions on the roof of the third ventricle and avoid using cautery in this area to prevent thermal damage to forniceal bundles. We use the swiveling technique and grasp the cyst wall with rotation to wrap it with tension and apply gentle pulling to remove it in one piece. We did not experience any findings of apparent forniceal bundle damage in our patients. There was only one patient with short-term memory loss, which resolved in the sixth-month follow-up period.

In addition, our experience showed that a wide working channel allows using sizable forceps, scissors, and radiofrequency coagulation probes; an angled view of 20°–30° and the shortest possible endoscope length are essential for good results. For large cysts or those located posteriorly, access can be enhanced by extending the opening of the foramen of Monroe through the choroidal fissure posteriorly (20). Moreover, neuronavigation plays a significant role in planning the location of the burr hole. Neuronavigation and ultrasound may be used for a ventricular puncture, especially for patients with slit ventricles (21).

### Comparison with microsurgery

Since 1983, microsurgical approaches to CCs, such as transcallosal, transcortical, or interhemispheric approaches, have been replaced by endoscopic techniques (22, 23). Compared with microsurgery, endoscopic resections have lower complication rates. The incidence rates of postoperative seizures, venous infarctions, and intracerebral hematomas are considerably lower (24, 25). According to reviews and meta-analyses, the overall morbidity rate is approximately 16.3% after microsurgery and 10.5% after the endoscopic approach for CCs. On the other hand, some studies reported higher total resection rates for microsurgery than for endoscopic surgery (97%–58%) (13, 26, 27). Eshra et al. reported 75% of the total resection rate in 16 patients operated on by a full-endoscopic approach without recurrence. The overall morbidity was significantly higher for microsurgery (19%–9%), and the rates of complications such as infection, seizures, and infarctions were much lower in the endoscopic groups (28). According to a meta-analysis of 1,278 patients with CCs, the microsurgical approach had a significantly higher total resection rate, lower recurrence rate, and lower reoperation rate than the endoscopic approach. The morbidity rate was lower for the endoscopic group. There was no significant difference in the mortality rate (1.4% vs. 0.6%) between the approaches (13). In our study, one patient had postoperative meningitis, and one had uncontrolled bleeding. No recurrence was detected in the follow-up period, hence no reoperation.

Greenlee et al. reported 35 consecutive patients with colloid cysts treated by endoscopic surgery in their retrospective study. The median follow-up period was 88 months, with only one asymptomatic radiological recurrence (29). In addition, Sribnick et al. reported only one recurrence in their prospective cohort study with endoscopically treated 56 patients with CCs in long-term follow-up (30). Overall, our study showed high total resection and low complication rates for full-endoscopic removal of third ventricle colloid cysts.

### Shunt dependency

Ventriculoperitoneal shunt (VPS) dependency was also lower after endoscopic removal, which was attributed to a minor disturbance to the ventricular wall (range, 3.5%–10%) (31). In a meta-analysis, there was no significant difference in the shunt dependency rates (6.2% vs. 3.9%) between microsurgical and endoscopic groups (13). In our study, persisting hydrocephalus rates were low. Therefore, we did not perform a routine septostomy or third ventriculostomy. Further studies with higher patient numbers can explore the effect of these procedures on patients with CCs that presented with hydrocephalus. In our study, of 13 patients that presented with hydrocephalus, only one that experienced uncontrolled bleeding during surgery required a permanent VPS.

### Limitations

The small sample size and the retrospective nature of the study are among the limitations. Patients did not undergo routine preoperative and postoperative systematic neurocognitive testing. In the period where the study was conducted, there were no microsurgically treated patients with CCs. Therefore, a comparison could not be made. Furthermore, the follow-up period was short in our study. An annual outpatient clinic control after the sixth month MRI is suggested for the patients. However, most patients did not attend after two years of follow-up. The sixth-month MRI control did not show any recurrence in CC. However, if followed up longer, recurrences might have been encountered.

The full-endoscopic system needs to be mastered by surgeons. But when recognized, there were no further technical disadvantages.

## Conclusion

Full-endoscopic approach for third ventricle colloid cyst removal with the assistance of neuronavigation and ultrasound is a feasible technique. Cyst aspiration followed by grasping and rotational maneuver for the cyst wall provides total removal with the resolution of the obstruction if present and relief of symptoms. In the literature, the swiveling technique was described once in a case report. Our study is the first case series to show that the technique is effective with low recurrence and complication rates.

## Data Availability

The raw data supporting the conclusions of this article will be made available by the authors, without undue reservation.

## Supplementary material

SUPPLEMENTARY VIDEO S1

The maneuvers performed during the surgery. The ventricular puncture is performed under ultrasound guidance. Aspiration and the dissection of the colloid cyst and the swiveling technique can be appreciated in the simultaneous recording of the endoscopic view and the surgeon’s hands.
