# Corrigendum: Full-endoscopic removal of third ventricular colloid cysts: technique, results, and limitations

**DOI:** 10.3389/fsurg.2023.1234433

**Published:** 2023-06-23

**Authors:** Tugrul Cem Unal, Altay Sencer, Ilyas Dolas, Cafer Ikbal Gulsever, Duran Sahin, Duygu Dolen, Musa Samet Ozata, Metehan Ozturk, Yavuz Aras, Aydin Aydoseli

**Affiliations:** Department of Neurosurgery, Istanbul Faculty of Medicine, Istanbul University, Istanbul, Turkey

**Keywords:** colloid cyst, full-endoscopic surgery, hydrocephalus, neuroendoscopy, swiveling technique

A corrigendum on Full-endoscopic removal of third ventricular colloid cysts: technique, results, and limitations By Unal TC, Sencer A, Dolas I, Gulsever CI, Sahin D, Dolen D, Ozata MS, Ozturk M, Aras Y and Aydoseli A. (2023) Front. Surg. 10:1174144. doi: 10.3389/FSURG.2023.1174144

In the published article, there was an error.

A correction has been made to Materials and Methods, ‘Patient population’, Paragraph Number 1. This sentence previously stated:

“This study was performed by the ethical standards of the Institutional Review Board of …. The data of 21 patients who underwent an operation at … between 2008 and 2019 by the same surgical team using the full-endoscopic approach were evaluated retrospectively.”

The corrected sentence appears below:

“This study was performed by the ethical standards of the Institutional Review Board of Istanbul University, Faculty of Medicine. The data of 21 patients who underwent an operation at Istanbul University, Faculty of Medicine, Department of Neurosurgery between 2008 and 2019 by the same surgical team using the full-endoscopic approach were evaluated retrospectively.”

The authors apologize for this error and state that this does not change the scientific conclusions of the article in any way. The original article has been updated.

